# FOXI3 promotes migration and proliferation in prostate cancer bone metastases, modulated by FGF8

**DOI:** 10.3389/fonc.2026.1819598

**Published:** 2026-06-18

**Authors:** Angana Mukherjee, Daniel P. Hollern, William A. Byrd, Tyeler S. Rayburn, Oluwasina G. Williams, Carrie Knight, Clayton C. Yates, Jacqueline D. Jones

**Affiliations:** 1 Department of Biological & Environmental Sciences, College of Science and Engineering, University, Troy, AL, United States; 2 The Salk Institute, La Jolla, CA, United States; 3 Downstate-One Brooklyn Health, Brooklyn, NY, United States; 4 Department of Pathology, The University of Alabama at Birmingham, Birmingham, AL, United States; 5 Department of Pathology, Johns Hopkins School of Medicine, Baltimore, MD, United States; 6 Department of Chemistry and Physics, College of Science and Engineering, Troy University, Troy, AL, United States

**Keywords:** bone metastasis (BM), Prostate Cancer, FOXI3 Gene, Osteomimicry, Cancer Cell Migration, Transcription Factor (TF), FGF8

## Abstract

Studies have shown that all men who die from prostate cancer exhibit bone involvement, highlighting the clinical significance of bone metastases. Previously, we demonstrated that prostate cancer exhibits elevated expression of *FOXI3;* a transcription factor critical to bone development. However, its functional role in prostate cancer progression remains unexplored. In this study, we explored FOXI3 expression in human prostate cancer tissues and cell lines. Immunohistochemical analysis revealed a significant association between FOXI3 expression, tumor pathology, and increasing tumor grade. Analysis of publicly available gene expression data from prostate cancer patients showed that *FOXI3* is markedly elevated in bone metastases and strongly correlates with *FGF8*, suggesting a potential bone-specific regulatory interaction between these factors. Consistent with this, treatment of bone-derived prostate cancer cells with FGF8 increased *FOXI3* RNA expression, whereas no such effect was observed in a brain-metastatic cell line. Further, we demonstrated that the FGF8–FOXI3 axis regulates bone-derived prostate cancer cell migration and proliferation in a *FOXI3* dependent manner. Together, our findings demonstrate a pro-metastatic role of *FOXI3* in prostate cancer progression.

## Introduction

Prostate cancer is the most commonly diagnosed invasive solid tumor in men worldwide, representing approximately 7-8% of all cancers, with an estimated 375, 000 deaths reported annually (1). Even though only 5-7% of prostate cancer patients exhibit metastasis to distant sites, the majority of these patients ultimately succumb to the disease (2). In particular, prostate cancer metastasis to bone is associated with significantly reduced survival and poor prognosis (2) (3). Therefore, it is critical to understand the mechanisms and potential targets involved in prostate cancer metastasis and colonization of the bone.

Although the exact mechanisms underlying the sequential process of prostate cancer metastasis to bone are unclear, studies have shown that the abundance of growth factors in the bone microenvironment primes the cancer cells to proliferate and flourish (4). The bone marrow microenvironment consists of a heterogeneous population of hematopoietic stem cells, osteoblasts, osteoclasts, bone stromal cells, bone transcription factors, and growth factors such as transforming growth factor beta (TGFβ), fibroblast growth factors (FGFs), insulin-like growth factors (IGFs), and bone morphogenetic proteins (BMPs) (5–8). Not surprisingly, many of these factors exhibit overlapping roles in both bone remodelling during early development and solid tumor progression (9–15). Notably, the forkhead family transcription factor forkhead box I3 (FOXI3) has been reported to play a crucial role during early bone development (16–20). Similarly, FGF8, a member of the FGF family, plays important roles in developmental signaling and skeletal biology (21, 22). In addition to its role in development, FGF8 has been implicated in tumor progression, and its overexpression in prostate cancer is associated with advanced disease and reduced patient survival (23). Developmental studies have further linked FOXI3 to FGF signaling, including FGF8 (16), supporting a potential relationship between these pathways that may extend from developmental processes to cancer progression.

In previous work, we highlighted the role of *FOXI3* in various developmental processes and noted many of them also influence tumor progression (24). We observed elevated *FOXI3* mRNA in a limited number of The Cancer Genome Atlas (TCGA) breast cancer bone metastasis samples and found that *FOXI3* mRNA expression increased with tumor stage in TCGA prostate cancer samples (24). These data suggested that *FOXI3* may have important roles in tumor progression and bone metastasis. In this study, we expanded our analysis of prostate cancer patients and combined computational studies and functional studies in bone-derived prostate cancer cells, showing that *FOXI3* mRNA mediates metastatic phenotypes and demonstrating a potential mechanistic linkage to a key upstream growth factor, FGF8.

## Materials and methods

### Cell culture, antibodies and reagents

Human prostate cancer cell lines DU-145, PC-3, and C4-2B were a gift from Dr. Laurie McCauley (University of Michigan, Ann Arbor) or purchased from ATCC. Cells were grown in DMEM with 10% FBS (Gibco) and 1% penicillin/streptomycin (Invitrogen) at 37 °C in 5% CO_2_ and were routinely confirmed to be mycoplasma-free (25).

### Immunohistochemistry

Prostate cancer tissue microarrays (TMAs) were obtained from TissueArray.Com LLC and UB Biolab (TMA# PRO811a and Pro162-02). Immunohistochemistry (IHC) was performed using the ABC kit (Vector Labs) and anti-FOXI3 antibody (Abcam#ab81824) following established protocols (26). Stained samples were blindly scored for cytoplasmic and nuclear FOXI3 intensity (0–4+) and the percentage of cells at each intensity was quantified using NIS Elements (Nikon).

### Quantitative RT-PCR

Total RNA was extracted from cultured cells using TRIzol reagent (Invitrogen). cDNA was synthesized from 1 µg RNA using a commercial reverse transcription kit. qRT-PCR was performed using TaqMan gene expression assays (Thermo Fisher Scientific) with FOXI3 (Hs03645828_s1; cat#4351372) and HPRT1 (Hs02800695_m1, cat# 4331182) as the endogenous control. PCR cycling conditions consisted of 95 °C for 2 min followed by 40 cycles of 95 °C for 15 s and 60 °C for 1 min. Primer and probe sequences are proprietary. Relative expression was calculated using the 2^-ΔΔCt method.

### RNA interference experiments

Cells were seeded in 10 cm plates to achieve approximately 60–70% confluence at the time of transfection. After 24 hours, cells were transfected with *FOXI3* specific or control siRNA (Origene) using Lipofectamine 2000 (Invitrogen), according to the manufacturer’s instructions. Post 24 hours of transfection, FOXI3 knockdown was confirmed by qRT-PCR, and only samples with >75% knockdown were used for experiments.

### Trypan blue assay

Trypan blue (Life Technologies) exclusion assay was used to determine cell viability. Cells were seeded at a density of 1 × 10^5^ cells per well in 12-well plates and allowed to adhere overnight in 1 mL of reduced-serum medium (5% FBS). The following day, cells were treated with varying FGF8 concentrations (0, 10, or 100 ng/mL) for 24 hours, trypsinized, mixed 1:1 with Trypan blue, and viable cells were counted using a Countess II automated cell counter (Thermo Fisher Scientific).

### Migration assay

siFOXI3 or siControl-treated, or untreated cells were seeded at a density of 3 × 10^5^ cells per well in 6-well plates, allowed to reach approximately 70%–80% confluence (~24 hours post-transfection), and serum-starved overnight in Opti-MEM medium to minimize proliferation (27, 28). A linear scratch was introduced in the cell monolayer using a sterile pipette tip following established wound healing assay protocols (27, 28). Cells were then treated with FGF8 (0, 10, or 100 ng/mL) in dialyzed, serum-reduced medium to further limit the contribution of exogenous growth factors. All experimental conditions were performed in parallel under identical conditions, including cell density, media composition, and treatment duration. Images were captured at 0 and 24 hours, and migration was quantified as the percentage reduction in the scratch area relative to baseline, with comparisons made across treatment groups using consistent measurement parameters.

### Invasion assays

Cancer cell invasiveness was determined using 24-well Transwell inserts (8 µm pores; BD Biosciences) following the manufacturer’s protocol. Matrigel-coated inserts were seeded with 6 × 10^4^ cells and treated with 100 ng/mL FGF8 in serum-free media with 10% FBS in the lower chamber as a chemoattractant. After 24 hours, non-invasive cells were removed, and invading cells on the underside were fixed in 100% methanol, stained with 0.1% crystal violet, and counted in three random 10× fields.

### Statistical and genomics analysis

Statistical analyses were performed using GraphPad Prism v5.0 (GraphPad, La Jolla, CA). ANOVA and independent Student’s *t*-test were used to determine statistical differences between experimental and control values. *P* values <0.05 were considered statistically significant. Gene expression and copy number analyses were performed using publicly available datasets of primary and metastatic prostate cancer samples (29, 30). Processed and normalized gene expression and copy number data were accessed through the cBioPortal for Cancer Genomics (31, 32). Analyses were performed using the cBioPortal’s built-in tools, including comparison of gene expression levels, assessment of copy number alterations, and correlation analyses between *FOXI3* and *FGF8* across sample groups.

## Results

### Increased FOXI3 expression is associated with high- grade prostate tumors

**Figure 1:**
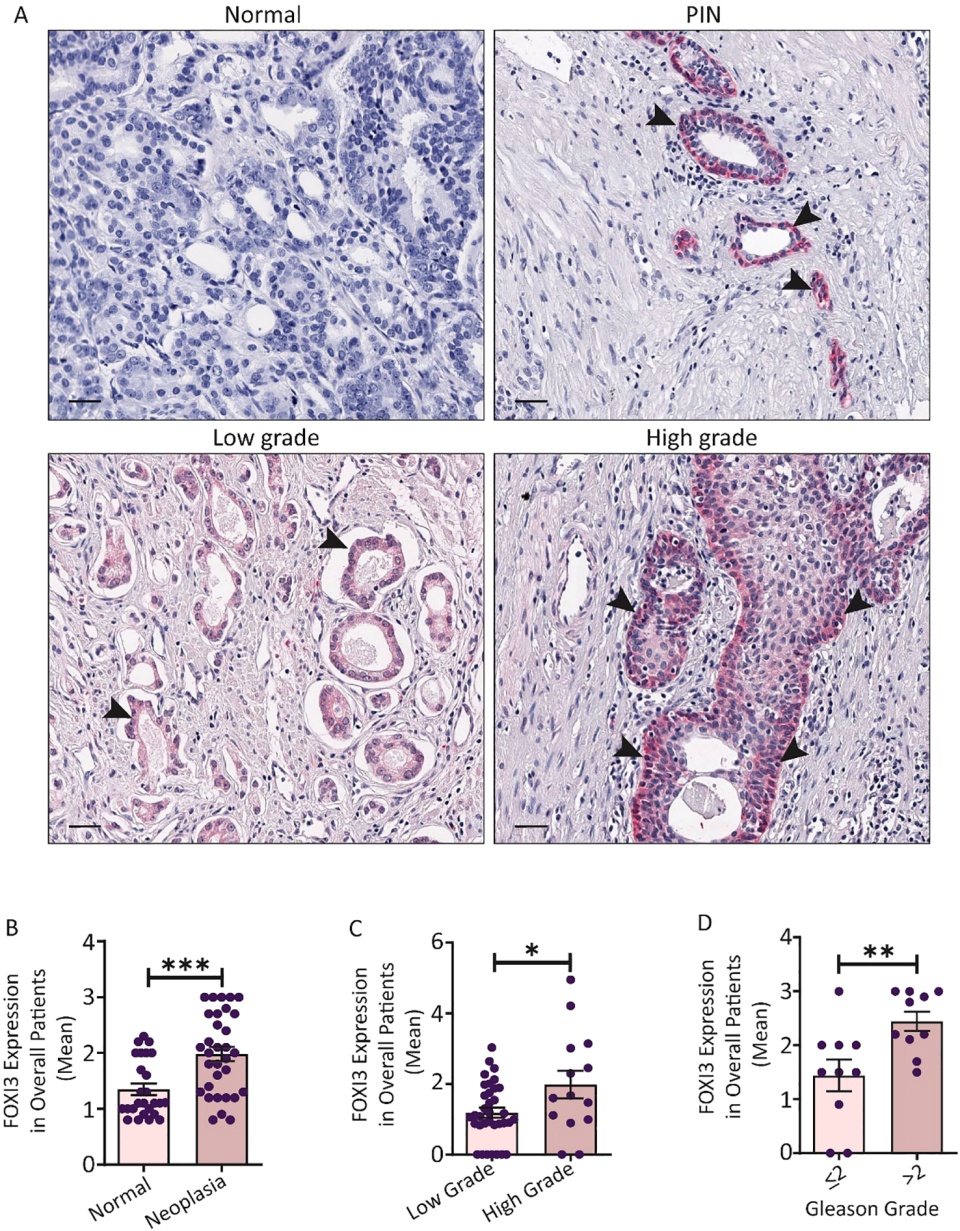
FOXI3 expression in human prostate cancer specimens. **(A)** Representative images show FOXI3 expression from IHC analyses of prostate cancer tissue microarrays (TMAs) performed in this study (normal = 26, PIN = 32, low-grade = 35, high-grade = 14, Gleason ≤2 = 10, Gleason >2 = 10). Arrows indicate positive cells. Total magnification is 10X. PIN is Prostatic intraepithelial neoplasia. **(B–D)** FOXI3 staining intensity was quantified computationally for each specimen and plotted according to pathology type, tumor grade and Gleason grade (*P=0.0191, **P=0.0097, ***P=0.0004).

To evaluate FOXI3 protein expression and its association with prostate tumor grade, we performed IHC on TMAs comprising samples from a cohort of prostate cancer patients with varying tissue pathology, with analyzable cases included in the downstream analysis (**Figure 1A**, **Supplementary Table S1**). Normal prostate tissues displayed minimal FOXI3 staining, consistent with low or near-background expression, while prostatic intraepithelial neoplasia (PIN) exhibited FOXI3 expression in the basal cell layer of ducts, suggesting early upregulation during tumorigenesis (**Figures 1A, B**). A comparison of FOXI3 expression across 91 patient tumor samples revealed significantly higher FOXI3 levels in grade 3–4 tumors compared with grade 1–2 tumors (**Figures 1A, C**; **Supplementary Table S1**). In a separate analysis, we stratified 32 patient samples based on Gleason grade—a widely used clinical metric of prostate cancer aggressiveness. Tumors with Gleason grades 3 to 5 exhibited significantly higher FOXI3 expression than those with Gleason grades 1 or 2 (**Figures 1A, D**, **Supplementary Table S1**). Collectively, these results suggest that elevated FOXI3 expression correlates with higher tumor grade and may serve as a marker of aggressive prostate cancer.

### FOXI3 expression is elevated with prostate bone metastasis and FGF8 expression.

**Figure 2:**
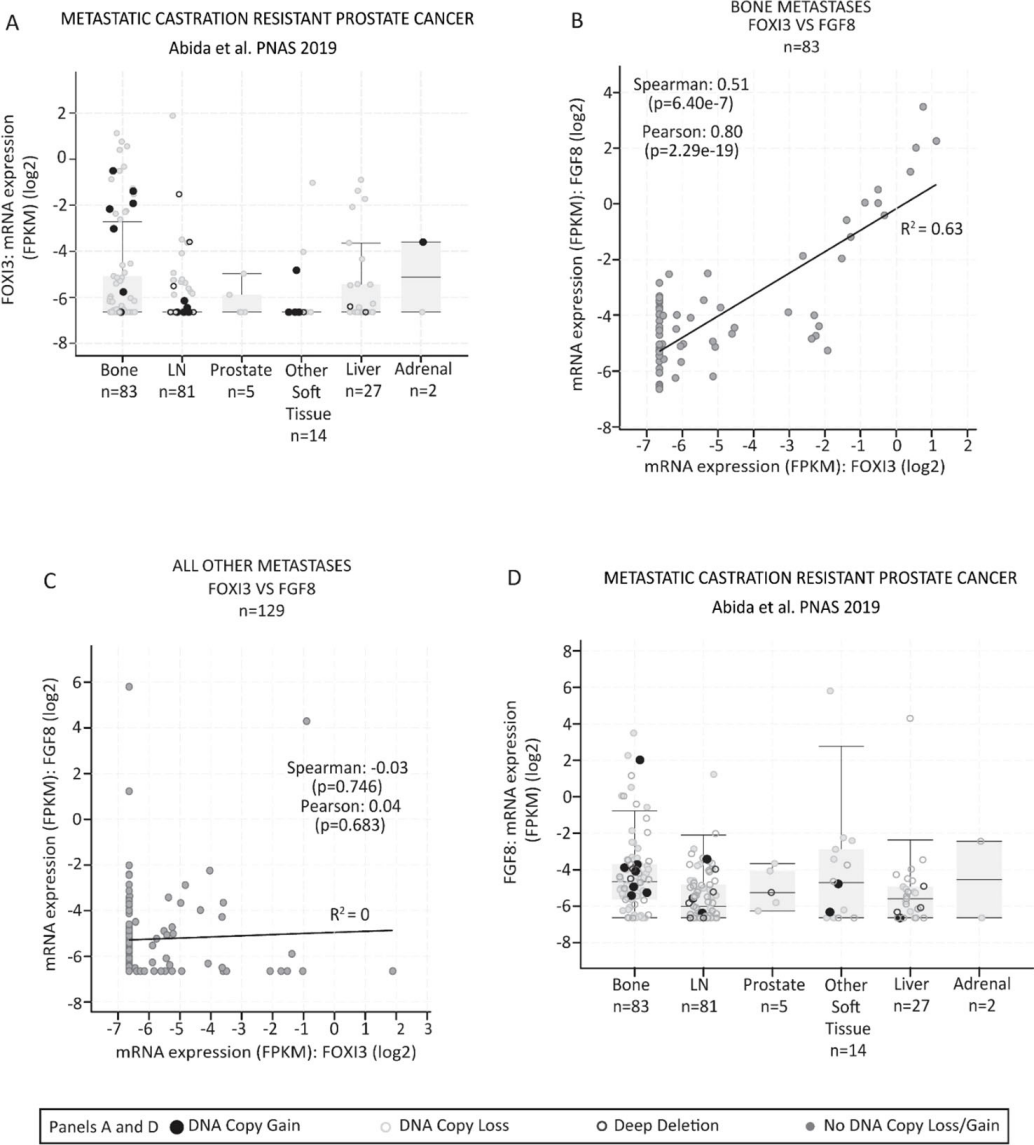
*FOXI3* expression and correlation with *FGF8* in prostate cancer metastasis. Gene expression and copy number analyses were performed using primary and metastatic patient samples from published and publicly available dataset (Abida et al., 2019) (25). **(A)** Processed, normalized, *FOXI3* gene expression and copy number across metastatic sites was graphed to demonstrate *FOXI3* expression patterns. **(B)** Spearman and Pearson correlation analysis show a significant correlation between *FOXI3* and *FGF8* in bone metastasis. **(C)** No significant correlation between *FOXI3* and *FGF8* was observed in non-bone metastatic sites. **(D)** *FGF8* mRNA expression in prostate cancer metastasis, including bone.

Building on the IHC data demonstrating elevated FOXI3 protein expression in high-grade prostate cancer, we hypothesized that FOXI3 expression may also correlate with metastatic progression. To test this, we analyzed gene expression data from two published studies featuring distant prostate cancer metastases (29, 30). While primary tumors showed a broad range of *FOXI3* mRNA levels, metastatic lesions generally exhibited higher expression, with bone metastases showing the highest *FOXI3* levels (**Figure 2A**; **Supplementary Figures S1A, B**). Notably, both primary and metastatic samples with the highest *FOXI3* expression often exhibited copy number gains at the FOXI3 locus, which were more common in bone metastases than in primary tumors (**Figure 2A**). Together, these data suggest that FOXI3 may contribute to bone-specific metastatic progression in prostate cancer and that copy number amplification could be a potential mechanism driving its overexpression in these lesions.

Because FOXI3 acts as a transcription factor, we next analyzed patient datasets to identify genes whose expression strongly covaries with *FOXI3* expression, aiming to uncover potential upstream regulators that may serve as therapeutic targets for modulating *FOXI3* activity. Our analysis revealed a significant correlation between *FOXI3* and *FGF8* expression in tumors with bone metastases (**Figure 2B**, **Supplementary Figure S1C**); but not in other metastatic sites (**Figure 2C**). Moreover, similar to FOXI3, bone metastasis samples with high FGF8 expression also exhibited increased FGF8 copy number gains (**Figure 2D**). Together, these analyses suggest a potential functional link between FOXI3 and FGF8 that may be particularly relevant for prostate cancer progression in the bone microenvironment.

### FOXI3 is overexpressed in prostate cancer bone metastatic cells and regulated by FGF8

**Figure 3:**
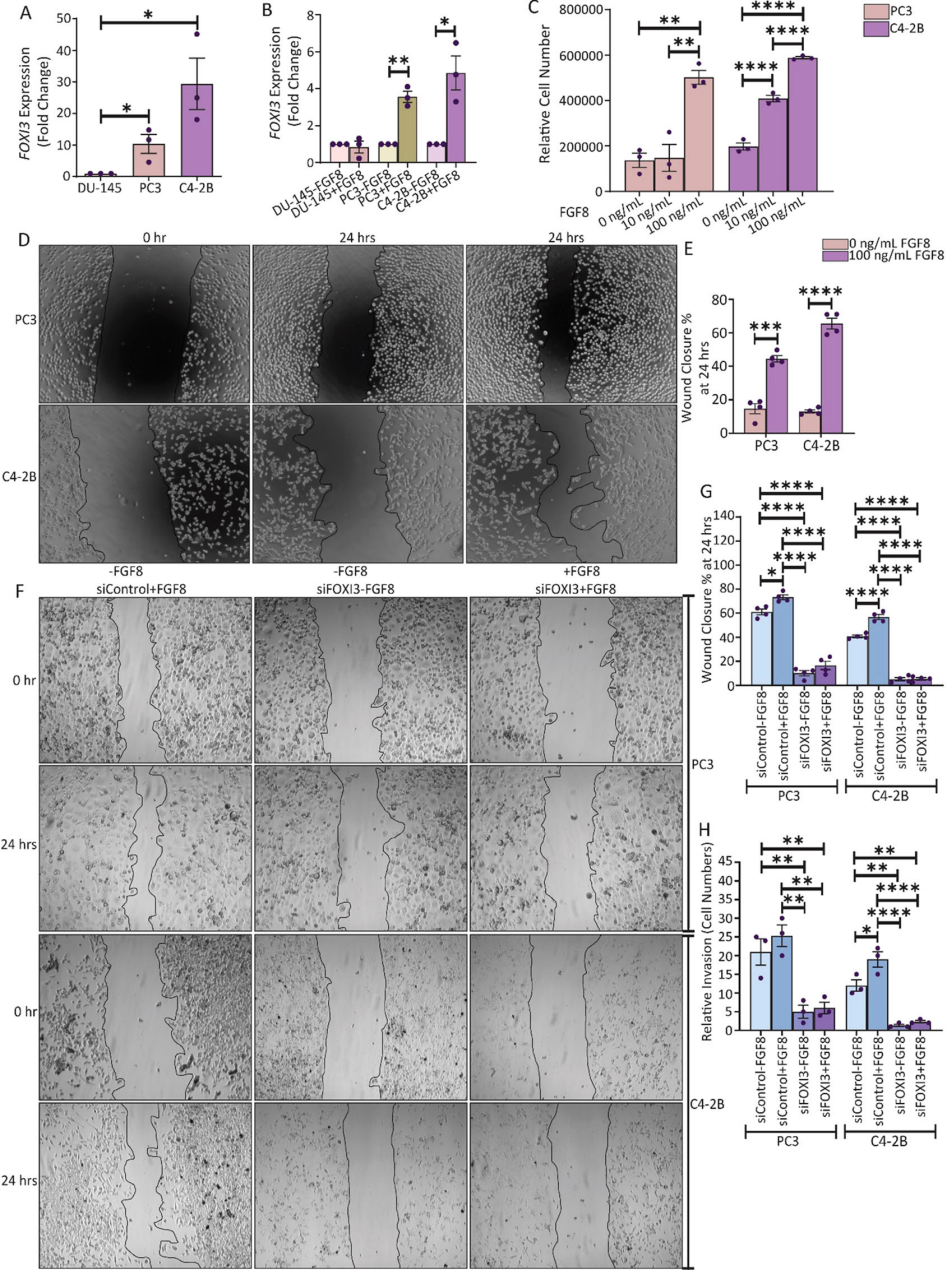
FGF8 elevates *FOXI3* expression and is required for FGF8-induced proliferation, migration, and invasion in bone-derived prostate cancer cell lines. **(A)** DU-145, PC3, and C4-2B prostate cancer cell lines were analyzed for *FOXI3* mRNA levels by quantitative RT-PCR (n=3 ± SE). HPRT1 was used as the endogenous control, and data was normalized to the brain tropic DU-145 cell line (^*^P= 0.0363, ^*^P= 0.0250). **(B)** DU-145, PC3, and C4-2B prostate cancer cells were treated with 100 ng/mL FGF8, respectively, and *FOXI3* mRNA expression was analyzed after 24 hours by quantitative RT-PCR. GAPDH was used as the endogenous control. Data was normalized to the respective untreated cells line (n=3 ± SE) (^*^P= 0.0138, ^**^P= 0.0011). **(C)** PC3 and C4-2B cells were treated with increasing concentrations of FGF8 (0ng/mL, 10 ng/mL, and 100 ng/mL) and after 24 hours cell viability was analyzed by trypan blue assay (n=3 ± SE) (PC: ^**^P=0.0016, ^**^P=0.0019; C4-2B: ^****^P<0.0001). **(D, E)** Post 24 hours of treatment with 100 ng/mL FGF8, a scratch-wound assay was performed on PC3 and C4-2B cells, respectively. Images were captured at a total magnification of 100X. Cell migration quantification was performed by measuring the distance between 3 random points within the wound edge in three replicate experiments. Data was normalized to 0 hours (n=4 ± SE) (PC: ^***^P=0.0002; C4–2B: ^****^P<0.0001). **(F, G)** 48 hours post transfection with siRNA against *FOXI3*, PC3, and C4-2B cells were treated with 100 ng/mL FGF8, and a scratch-wound assay was performed, respectively. Images were captured at a total magnification of 100X. Data was normalized to 0 hours (PC: ^*^P=0.0119, ^****^P<0.0001; C4-2B: ^****^P<0.0001) (n=3 ± SE). **(H)** siFOXI3 transfected PC3 and C4-2B cells were plated on matrigel-coated filters, and an invasion assay was performed. Invasive cells were stained with crystal violet, and enumerated. (n=3 ± SE) (PC: ^**^P=0.0056, ^**^P=0.0082, ^**^P=0.0013, ^**^P=0.0018; C4-2B: ^*^P=0.0138, ^**^P=0.0011, ^**^P=0.0021, ^****^P<0.0001).

To identify suitable models for functional studies, we compared *FOXI3* expression in prostate cancer cell lines from bone metastases (PC3 and C4-2B) with brain-metastatic DU-145 cells (33, 34). qRT-PCR analysis showed that *FOXI3* expression was significantly upregulated in the bone-derived lines, 4-fold higher in PC-3 and 30-fold higher in C4-2B cells compared to brain-derived prostate cancer cell, DU-145 (**Figure 3A**), reflecting the increased *FOXI3* levels observed in patient bone metastases.

Given the positive correlation between *FOXI3* and *FGF8* in prostate cancer bone metastasis (**Figure 2B**), and the known role of FGF8 in modulating FOXI3 during early development (16), we hypothesized that FGF8 regulates FOXI3 activity in bone-metastatic prostate cancer. To test this, we treated prostate cancer cell lines with FGF8 and measured *FOXI3* expression. Consistent with patterns observed in patient metastasis data (**Figures 2B, D**), FGF8 selectively increased *FOXI3* levels in bone-derived PC3 and C4-2B cells, but had no effect on brain-derived DU-145 cells (**Figure 3B**). In addition, qRT-PCR analysis revealed a 30-fold and 80-fold increase in *FOXI3* mRNA expression in PC3 and C4-2B cells, respectively, compared to DU-145 following FGF8 treatment (**Supplementary Figure S2A**), exceeding the baseline *FOXI3* expression observed in untreated cells (**Figure 3A**). Together, these results indicate that FGF8 regulates *FOXI3* expression in a cell type–specific manner, particularly in bone-derived prostate cancer cells.

### FOXI3 and FGF8 promotes proliferation and migration in bone-metastatic prostate cancer cells

While our data showed that FGF8 regulates *FOXI3* expression in bone-derived prostate cancer cells, it remained unclear whether this interaction contributes to cancer cell progression. To address this, we treated PC3 and C4-2B cells with increasing concentrations of FGF8 (0, 10, and 100 ng/mL), and viable cell numbers were assessed after 24 hours. In PC3 cells, treatment with 100 ng/mL resulted in a significant increase in viable cell number (**Figure 3C**), while C4-2B cells exhibited a dose-dependent response with significant increases in cell number observed at both 10 ng/mL and 100 ng/mL FGF8 (**Figure 3C**). To assess the effect of FGF8 on prostate cancer cell migration, we performed a scratch wound assay using PC3 and C4-2B cells. FGF8 treatment significantly increased the migration rate in both bone-derived lines PC3 and C4-2B (**Figures 3D, E**), whereas DU-145 cells, which express low levels of *FOXI3*, showed no significant change in the migration rate with FGF8 (**Supplementary Figures S2B, C**). These results suggest that FGF8 promotes a more aggressive, migratory phenotype specifically in *FOXI3*-high prostate cancer cells.

To determine whether these FGF8-driven phenotypes require FOXI3, we performed siRNA-mediated knockdown of *FOXI3* in PC3 and C4-2B cells (**Supplementary Figure S2D**). Reduced *FOXI3* expression markedly diminished FGF8-induced cell number (**Supplementary Figure S2E**) and also significantly impaired migration and invasion in both cell lines, even without FGF8 stimulation (**Figures 3F–H**). These findings show that *FOXI3* not only mediates FGF8-induced behaviors but also independently drives migratory and invasive phenotypes, underscoring the functional significance of the FOXI3-FGF8 axis in prostate cancer progression.

Together, results demonstrate that FGF8-mediated aggressive phenotypes are FOXI3-dependent, and that FOXI3 itself controls key processes required for metastatic progression. When combined with our computational analyses of patient datasets, these findings implicate FOXI3 as a key regulator of prostate cancer malignancy and a likely promoter of bone-tropic metastatic behavior.

## Discussion

In this study, we integrated genomic datasets with immunohistochemical analysis of prostate tumor microarrays to investigate the role of FOXI3 in prostate cancer, with a focus on bone metastasis. Building on our earlier work implicating FOXI3 in cancer biology (24), we found that FOXI3 expression increases with tumor grade, supporting its association with aggressive disease. Clinical datasets further revealed co-elevated expression of *FOXI3* and the bone-associated growth factor FGF8, specifically in bone metastases. Our functional assays demonstrated that this correlation has biological significance: FGF8 upregulates *FOXI3* in bone-derived prostate cancer cells, promoting increased cell growth and migration. To our knowledge, this is the first direct evidence of a functional role for FOXI3 in cancer, establishing it as a potential candidate effector in prostate cancer progression to bone.

Analysis of clinical datasets also revealed that a subset of prostate cancer patients exhibited copy number gains at the FOXI3 locus, with some also showing FGF8 amplifications. In both cases, these alterations were associated with increased gene expression and occurred more frequently in bone metastases than in primary tumors. Given that copy number changes are established drivers of metastatic progression (35, 36), our findings suggest that FOXI3 may similarly function as a driver in prostate cancer.

We also observed a significant correlation between FOXI3 and FGF8 in patients with prostate cancer bone metastases. This relationship was functionally validated by *in vitro* experiments, which showed that FGF8 promotes *FOXI3* expression, leading to increased proliferation and migration of prostate cancer cells derived from bone metastases. Notably, this correlation was not observed in other metastatic sites, suggesting that the FGF8–FOXI3 axis contributes specifically to bone tropism. These findings highlight the need for future *in vivo* studies to define FOXI3 downstream targets and to identify the cellular sources of FGF8, which may uncover therapeutic strategies to block bone metastasis or eradicate disseminated tumor cells. The association of FOXI3 with higher tumor grade, together with functional evidence showing its role in cell proliferation, migration, and invasion, supports the idea that tumor clones with elevated *FOXI3* and *FGF8*—likely resulting from genomic amplification —may have an enhanced capacity to metastasize to bone, underscoring the need for *in vivo* studies to define FOXI3 downstream targets and clarify the cellular sources of FGF8. Such work could inform therapeutic approaches aimed at blocking bone metastasis or eradicating disseminated tumor cells.

**Figure 4:**
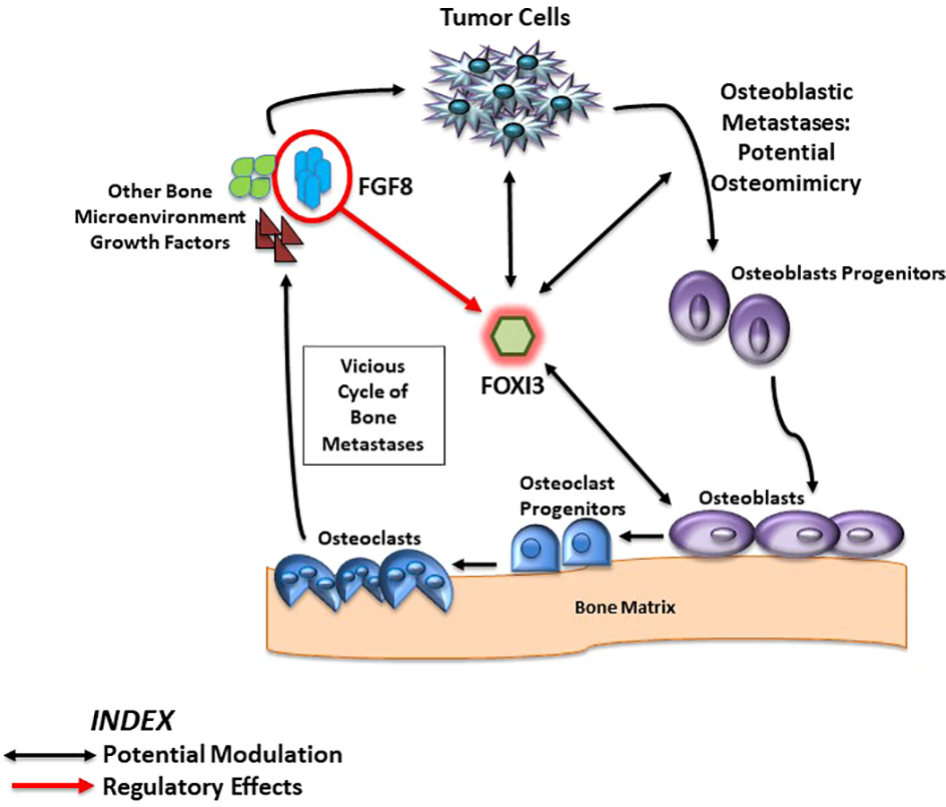
Proposed model depicting the role of FOXI3 in prostate cancer bone metastasis. Metastatic prostate cancer cells may adopt osteoblast-like features, leading to enhanced FOXI3 activity that contributes to bone remodeling. Within this context, FOXI3 may be further modulated by the bone microenvironment factor FGF8, which amplifies tumor proliferation and colonization, thereby promoting bone metastasis.

The observed copy number gains in FGF8 further suggest that tumor cells themselves may be a source of FGF8, consistent with earlier reports (23, 37). Developmental studies have linked FOXI3 to FGF signaling centers, with associated effects on FGF8 expression (16), supporting a potential conserved relationship between these pathways. However, given the developmental roles of both FOXI3 and FGF8 in bone biology (16–22), it is also plausible that the bone microenvironment, particularly osteoblasts, which are a known source of FGF8 (22), contributes to elevated FGF8 levels that potentially activate FOXI3 expression in tumor cells, thereby promoting cell growth and colonization. This aligns with the concept of osteomimicry, whereby prostate cancer cells adopt osteoblast-like programs to adapt to the bone niche (38–40). Given their shared developmental roles, prostate cancer cells may exploit FGF8-driven FOXI3 activation to sustain survival and expansion in bone (**Figure 4**).

In summary, this study provides the first patient-relevant evidence implicating FOXI3, in association with FGF8, in prostate cancer progression to bone. Supported by *in vitro* functional data, we show that *FOXI3* expression is closely linked to cell growth and migration of bone-derived prostate cancer cells. As a brief communication, our findings open several avenues for future investigation, including identifying FOXI3 target genes, defining bone-derived regulators of FOXI3, and elucidating its role in osteomimicry. Together, these studies will be critical for determining whether FOXI3 and FGF8 can be leveraged as therapeutic targets in advanced prostate cancer with bone metastases.

## Data Availability

The original contributions presented in the study are included in the article/**Supplementary Material**. Further inquiries can be directed to the corresponding author.

## References

[1] Siegel RL Miller KD Jemal A . Cancer statistics, 2024. CA: A Cancer J For Clin. (2024) 74:12–49. doi: 10.3322/caac.21820 38230766

[2] Baldessari C Pipitone S Molinaro E Cerma K Fanelli M Nasso C . Bone metastases and health in prostate cancer: from pathophysiology to clinical implications. Cancers. (2023) 15:1518. doi: 10.3390/cancers15051518 36900309 PMC10000416

[3] Posdzich P Darr C Hilser T Wahl M Herrmann K Hadaschik B . Metastatic prostate cancer—a review of current treatment options and promising new approaches. Cancers. (2023) 15:461. doi: 10.3390/cancers15020461 36672410 PMC9856730

[4] Olechnowicz SW Edwards CM . Contributions of the host microenvironment to cancer-induced bone disease. Cancer Res. (2014) 74:1625–31. doi: 10.1158/0008-5472.can-13-2645 24599133 PMC3966188

[5] Thobe MN Clark RJ Bainer RO Prasad SM Rinker-Schaeffer CW . From prostate to bone: key players in prostate cancer bone metastasis. Cancers. (2011) 3:478–93. doi: 10.3390/cancers3010478 21603150 PMC3096870

[6] Goode EA Wang N Munkley J . Prostate cancer bone metastases biology and clinical management. Oncol Lett. (2023) 25:163. doi: 10.3892/ol.2023.13749 36960185 PMC10028493

[7] Casimiro S Luis I Fernandes A Pires R Pinto A Gouveia AG . Analysis of a bone metastasis gene expression signature in patients with bone metastasis from solid tumors. Clin Exp Metastasis. (2012) 29:155–64. doi: 10.1007/s10585-011-9438-0 22120474

[8] Ren G Esposito M Kang Y . Bone metastasis and the metastatic niche. J Mol Med (Berlin Germany). (2015) 93:1203–12. doi: 10.1007/s00109-015-1329-4 26275789 PMC4636917

[9] Wu M Chen G Li Y-P . TGF-β and BMP signaling in osteoblast, skeletal development, and bone formation, homeostasis and disease. Bone Res. (2016) 4:16009. doi: 10.1038/boneres.2016.9 27563484 PMC4985055

[10] Dorey K Amaya E . FGF signalling: diverse roles during early vertebrate embryogenesis. Development. (2010) 137:3731–42. doi: 10.1242/dev.037689 20978071 PMC3747497

[11] Charoenlarp P Rajendran AK Iseki S . Role of fibroblast growth factors in bone regeneration. Inflammation Regeneration. (2017) 37:10. doi: 10.1186/s41232-017-0043-8 29259709 PMC5725923

[12] Hollern DP Swiatnicki MR Rennhack JP Misek SA Matson BC McAuliff A . E2F1 drives breast cancer metastasis by regulating the target gene FGF13 and altering cell migration. Sci Rep. (2019) 9:10718. doi: 10.1038/s41598-019-47218-0 31341204 PMC6656723

[13] Meng X Vander Ark A Lee P Hostetter G Bhowmick NA Matrisian LM . Myeloid-specific TGF-beta signaling in bone promotes basic-FGF and breast cancer bone metastasis. Oncogene. (2016) 35:2370–8. doi: 10.1038/onc.2015.297 26279296

[14] Chen G Deng C Li Y-P . TGF-β and BMP signaling in osteoblast differentiation and bone formation. Int J Biol Sci. (2012) 8:272. doi: 10.7150/ijbs.2929 22298955 PMC3269610

[15] Jiang H . Prostate cancer bone metastasis: molecular mechanisms of tumor and bone microenvironment. Cancer Manage Res. (2025) 17:219–37. doi: 10.2147/CMAR.S495169 39912095 PMC11796448

[16] Edlund RK Ohyama T Kantarci H Riley BB Groves AK . Foxi transcription factors promote pharyngeal arch development by regulating formation of FGF signaling centers. Dev Biol. (2014) 390:1–13. doi: 10.1016/j.ydbio.2014.03.004 24650709 PMC4013273

[17] Jussila M Aalto AJ Sanz Navarro M Shirokova V Balic A Kallonen A . Suppression of epithelial differentiation by Foxi3 is essential for molar crown patterning. Development. (2015) 142:3954–63. doi: 10.1242/dev.124172 26450968 PMC6517835

[18] Khatri SB Groves AK . Expression of the Foxi2 and Foxi3 transcription factors during development of chicken sensory placodes and pharyngeal arches. Gene Expression Patterns: GEP. (2013) 13:38–42. doi: 10.1016/j.gep.2012.10.001 23124078 PMC3562376

[19] Tassano E Jagannathan V Drögemüller C Leoni M Hytönen MK Severino M . Congenital aural atresia associated with agenesis of internal carotid artery in a girl with a FOXI3 deletion. Am J Med Genet Part A. (2015) 167a:537–44. doi: 10.1002/ajmg.a.36895 25655429 PMC4591042

[20] Thawani A Maunsell HR Zhang H Ankamreddy H Groves AK . The Foxi3 transcription factor is necessary for the fate restriction of placodal lineages at the neural plate border. Development. (2023) 150:dev202047. doi: 10.1242/dev.202047 37756587 PMC10617604

[21] Ornitz DM Itoh N . The fibroblast growth factor signaling pathway. Wiley Interdiscip Reviews: Dev Biol. (2015) 4:215–66. doi: 10.1002/wdev.176 25772309 PMC4393358

[22] Su N Jin M Chen L . Role of FGF/FGFR signaling in skeletal development and homeostasis: learning from mouse models. Bone Res. (2014) 2:14003. doi: 10.1038/boneres.2014.3 26273516 PMC4472122

[23] Dorkin TJ Robinson MC Marsh C Bjartell A Neal DE Leung HY . FGF8 over-expression in prostate cancer is associated with decreased patient survival and persists in androgen independent disease. Oncogene. (1999) 18:2755. doi: 10.1038/sj.onc.1202624 10348350

[24] Mukherjee A Hollern DP Williams OG Rayburn TS Byrd WA Yates C . A review of FOXI3 regulation of development and possible roles in cancer progression and metastasis. Front Cell Dev Biol. (2018) 6:69. doi: 10.3389/fcell.2018.00069 30018953 PMC6038025

[25] Jones J Wang H Karanam B Theodore S Dean-Colomb W Welch DR . Nuclear localization of Kaiso promotes the poorly differentiated phenotype and EMT in infiltrating ductal carcinomas. Clin Exp Metastasis. (2014) 31:497–510. doi: 10.1007/s10585-014-9644-7 24570268 PMC4065802

[26] Jones J Mukherjee A Karanam B Davis M Jaynes J Reams RR . African Americans with pancreatic ductal adenocarcinoma exhibit gender differences in Kaiso expression. Cancer Lett. (2017) 380:513–22. doi: 10.1016/j.canlet.2016.06.025 27424525 PMC5003655

[27] Cory G . Scratch-wound assay. Methods Mol Biol. (2011) 769:25–30. doi: 10.1007/978-1-61779-207-6_2 21748666

[28] Liang CC Park AY Guan JL . *In vitro* scratch assay: a convenient and inexpensive method for analysis of cell migration *in vitro*. Nat Protoc. (2007) 2:329–33. doi: 10.1038/nprot.2007.30 17406593

[29] Taylor BS Schultz N Hieronymus H Gopalan A Xiao Y Carver BS . Integrative genomic profiling of human prostate cancer. Cancer Cell. (2010) 18:11–22. doi: 10.1016/j.ccr.2010.05.026 20579941 PMC3198787

[30] Abida W Cyrta J Heller G Prandi D Armenia J Coleman I . Genomic correlates of clinical outcome in advanced prostate cancer. Proc Natl Acad Sci. (2019) 116:11428–36. doi: 10.1073/pnas.1902651116 31061129 PMC6561293

[31] Cerami E Gao J Dogrusoz U Gross BE Sumer SO Aksoy BA . The cBio cancer genomics portal: an open platform for exploring multidimensional cancer genomics data. Cancer Discov. (2012) 2:401–4. doi: 10.1158/2159-8290.CD-12-0095 22588877 PMC3956037

[32] Gao J Aksoy BA Dogrusoz U Dresdner G Gross B Sumer SO . Integrative analysis of complex cancer genomics and clinical profiles using the cBioPortal. Sci Signal. (2013) 6:pl1-pl1. doi: 10.1126/scisignal.2004088 23550210 PMC4160307

[33] Cunningham D You Z . *In vitro* and *in vivo* model systems used in prostate cancer research. J Biol Methods. (2015) 2:e17. doi: 10.14440/jbm.2015.63 26146646 PMC4487886

[34] Saranyutanon S Deshmukh SK Dasgupta S Pai S Singh S Singh AP . Cellular and molecular progression of prostate cancer: models for basic and preclinical research. Cancers. (2020) 12:2651. doi: 10.3390/cancers12092651 32957478 PMC7563251

[35] Pearlman A Upadhyay K Cole K Loke J Sun K Fineberg S . Robust genomic copy number predictor of pan cancer metastasis. Genes Cancer. (2018) 9:66. doi: 10.18632/genesandcancer.165 29725504 PMC5931251

[36] Siegel MB He X Hoadley KA Hoyle A Pearce JB Garrett AL . Integrated RNA and DNA sequencing reveals early drivers of metastatic breast cancer. J Clin Invest. (2018) 128:1371–1383. doi: 10.1172/jci96153 29480819 PMC5873890

[37] Ghosh AK Shankar DB Shackleford GM Wu K T'Ang A Miller GJ . Molecular cloning and characterization of human FGF8 alternative messenger RNA forms. Cell Growth Differentiation-Publication Am Assoc For Cancer Res. (1996) 7:1425–34. 8891346

[38] Koeneman KS Yeung F Chung LW . Osteomimetic properties of prostate cancer cells: a hypothesis supporting the predilection of prostate cancer metastasis and growth in the bone environment. Prostate. (1999) 39:246–61. doi: 10.1002/(sici)1097-0045(19990601)39:4<246::aid-pros5>3.0.co;2-u 10344214

[39] Rucci N Teti A . Osteomimicry: how tumor cells try to deceive the bone. Front Bioscience (Scholar Edition). (2010) 2:907–15. doi: 10.2741/s110 20515833

[40] Zhang X . Interactions between cancer cells and bone microenvironment promote bone metastasis in prostate cancer. Cancer Commun. (2019) 39:76. doi: 10.1186/s40880-019-0425-1 31753020 PMC6873445

