# Supplementary material for: FOXI3 promotes migration and proliferation in prostate cancer bone metastases, modulated by FGF8
Source: Front Oncol. 2026 Jun 18;16:1819598. doi: 10.3389/fonc.2026.1819598 (PMC13323151; doi:10.3389/fonc.2026.1819598)
Supplement: Supplementary file 1 [file DataSheet1.pdf]

Location	***Gender***	***Grade***	***Gleason Garde***	***Stage T***	***Stage N***	***Stage M***
A1	M	1	1	3	0	0
A2	M	1	1	2	0	0
A3	M	2	3	2	0	0
A4	M	2	2	4	1	1
A5	M	1	1	4	1	1
A6	M	2	2	2	0	0
A7	M	2	2	2	1	1
A8	M	2	3	3	1	0
A9	M	2	3	2	0	0
A10	M	2	4	2	0	0
B1	M	2	3	3	1	1
B2	M	3	4	2	0	0
B3	M	3	4	3	1	0
B4	M	3	4	3	0	0
B5	M	2--3	3--4	2	1	1
B6	M	2	3	2	0	0
B7	M	3	4	3	0	1
B8	M	2	3	2	1	1
B9	M	3	4	3	1	1
B10	M	3	4	2	1	1
C1	M	2	2	4	1	1
C2	M	1	1	3	0	0
C3	M	3	4	2	0	0
C4	M	3	4	2	0	1
C5	M	3	4	2	2	0
C6	M	4	5	2	0	0
C7	M	1	1	2	1	1
C8	M	4	5	4	1	1
C9	M	4	5	4	0	1
C10	M	4	5	2	0	0
D1	M	2	2	2	0	0
D2	M	3	4	2	0	0
D3	M	2--3	-	-	-	-
D4	M	3	-	-	-	-
D5	M	-	-	-	-	-
D6	M	-	-	-	-	-
D7	M	-	-	-	-	-
D8	M	-	-	-	-	-
D9	M	-	-	-	-	-
D10	M	-	-	-	-	-
E1	M	-	-	-	-	-
E2	M	-	-	-	-	-
E3	M	1	-	-	-	-
E4	M	-	-	-	-	-
E5	M	-	-	-	-	-
E6	M	-	-	-	-	-

E7	M	-	-	-	-	-
E8	M	-	-	-	-	-
E9	M	-	-	-	-	-
E10	M	-	-	-	-	-
F1	M	-	-	-	-	-
F2	M	-	-	-	-	-
F3	M	-	-	-	-	-
F4	M	-	-	-	-	-
F5	M	-	-	-	-	-
F6	M	-	-	-	-	-
F7	M	-	-	-	-	-
F8	M	-	-	-	-	-
F9	M	-	-	-	-	-
F10	M	-	-	-	-	-
G1	M	-	-	-	-	-
G2	M	-	-	-	-	-
G3	M	-	-	-	-	-
G4	M	-	-	-	-	-
G5	M	-	-	-	-	-
G6	M	-	-	-	-	-
G7	M	-	-	-	-	-
G8	M	-	-	-	-	-
G9	M	-	-	-	-	-
G10	M	-	-	-	-	-
H1	M	-	-	-	-	-
H2	M	-	-	-	-	-
H3	M	-	-	-	-	-
H4	M	-	-	-	-	-
H5	M	-	-	-	-	-
H6	M	-	-	-	-	-
H7	M	-	-	-	-	-
H8	M	-	-	-	-	-
H9	M	-	-	-	-	-
H10	M	-	-	-	-	-

PRO811a_US Biomax

Pathology
AD
AD
AD
AD
AD
AD
AD
AD
AD
AD
AD
AD
AD
AD
AD
AD
AD
AD
AD
AD
AD
AD
AD
AD
AD
AD
AD
AD
AD
AD
Small Ac
Small Ac
Metastatic AD
Metastatic AD
Hyperplasia
Hyperplasia
Hyperplasia
Hyperplasia
Hyperplasia
Hyperplasia
Hyperplasia
Hyperplasia
Hyperplasia AD
Hyperplasia
Hyperplasia
Hyperplasia

Hyperplasia
Hyperplasia
Hyperplasia
Hyperplasia
Hyperplasia
Hyperplasia
Hyperplasia
Hyperplasia
Hyperplasia
Hyperplasia
Hyperplasia
Hyperplasia
Hyperplasia
Hyperplasia
Chronic Inflammation
Chronic Inflammation
Chronic Inflammation
Chronic Inflammation
Chronic Inflammation
Chronic Inflammation
Cancer Adjacent Pr Tissue
Cancer Adjacent Pr Tissue
Cancer Adjacent Pr Tissue
Cancer Adjacent Pr Tissue
Cancer Adjacent Pr Tissue
Cancer Adjacent Pr Tissue
Pr Tissue
Pr Tissue
Pr Tissue
Pr Tissue
Pr Tissue
Pr Tissue
Pr Tissue (Smooth muscle)
Pr Tissue

Location	*Grade*	*Pathology*	**Pro162-02_US BIolab**
A1	2--3	Pr Cancer	
A2	2--3	Pr Cancer	
A3	1--2	Pr Cancer	
A4	1--2	Pr Cancer	
A5	2	Pr Cancer	
A6	2	Pr Cancer	
A7	2	Pr Cancer	
A8	2	Pr Cancer	
A9	2	Pr Cancer	
A10	2	Pr Cancer	
A11	2	Pr Cancer	
A12	2	Pr Cancer	
A13	2--3	Pr Cancer	
A14	2--3	Pr Cancer	
A15	2	Pr Cancer	
A16	2	Pr Cancer	
B1	3	Pr Cancer	
B2	3	Pr Cancer	
B3	2	Pr Cancer	
B4	2	Pr Cancer	
B5	2--3	Pr Cancer	
B6	2--3	Pr Cancer	
B7	3	Pr Cancer	
B8	3	Pr Cancer	
B9	2--3	Pr Cancer	
B10	2--3	Pr Cancer	
B11	2	Pr Cancer	
B12	2	Pr Cancer	
B13	2	Pr Cancer	
B14	2	Pr Cancer	
B15	2	Pr Cancer	
B16	2	Pr Cancer	
C1	2	Pr Cancer	
C2	2	Pr Cancer	
C3	2	Pr Cancer	
C4	2	Pr Cancer	
C5	2	Pr Cancer	
C6	2	Pr Cancer	
C7	2	Pr Cancer	
C8	2	Pr Cancer	
C9	2	Pr Cancer	
C10	2	Pr Cancer	
C11	1--2	Pr Cancer	
C12	1--2	Pr Cancer	
C13	2	Pr Cancer	
C14	2	Pr Cancer	

C15	2--3	Pr Cancer
C16	2--3	Pr Cancer
D1	2	Pr Cancer
D2	2	Pr Cancer
D3	2--3	Pr Cancer
D4	2--3	Pr Cancer
D5	-	Pr Cancer
D6	-	Pr Cancer
D7	-	Pr Cancer
D8	-	Pr Cancer
D9	-	Pr Cancer
D10	-	Pr Cancer
D11	-	Pr Cancer
D12	-	Pr Cancer
D13	-	Pr Cancer
D14	-	Pr Cancer
D15	-	Pr Cancer
D16	-	Pr Cancer
E1	-	Pr Cancer
E2	-	Pr Cancer
E3	2--3	Pr Cancer
E4	2--3	Pr Cancer
E5	2	Pr Cancer
E6	2	Pr Cancer
E7	1--2	Pr Cancer
E8	1--2	Pr Cancer
E9	-	Intrepithelial neoplasia
E10	-	Intrepithelial neoplasia
E11	-	Intrepithelial neoplasia
E12	-	Intrepithelial neoplasia
E13	-	Intrepithelial neoplasia
E14	-	Intrepithelial neoplasia
E15	-	Intrepithelial neoplasia
E16	-	Intrepithelial neoplasia
F1	-	Intrepithelial neoplasia
F2	-	Intrepithelial neoplasia
F3	-	Intrepithelial neoplasia
F4	-	Intrepithelial neoplasia
F5	-	Intrepithelial neoplasia
F6	-	Intrepithelial neoplasia
F7	-	Intrepithelial neoplasia
F8	-	Intrepithelial neoplasia
F9	-	Intrepithelial neoplasia
F10	-	Intrepithelial neoplasia
F11	-	Intrepithelial neoplasia
F12	-	Intrepithelial neoplasia
F13	-	Intrepithelial neoplasia

F14	-	Intrepithelial neoplasia
F15	-	Intrepithelial neoplasia
F16	-	Intrepithelial neoplasia
G1	-	Intrepithelial neoplasia
G2	-	Intrepithelial neoplasia
G3	-	Intrepithelial neoplasia
G4	-	Intrepithelial neoplasia
G5	-	Intrepithelial neoplasia
G6	-	Intrepithelial neoplasia
G7	-	Intrepithelial neoplasia
G8	-	Intrepithelial neoplasia
G9	-	Intrepithelial neoplasia
G10	-	Intrepithelial neoplasia
G11	-	Intrepithelial neoplasia
G12	-	Intrepithelial neoplasia
G13	-	Intrepithelial neoplasia
G14	-	Intrepithelial neoplasia
G15	-	Chronic Prostatitis
G16	-	Chronic Prostatitis
H1	-	Normal Gland
H2	-	Normal Gland
H3	-	Normal Gland
H4	-	Normal Gland
H5	-	Normal Gland
H6	-	Normal Gland
H7	-	Normal Gland
H8	-	Normal Gland
H9	-	Normal Gland
H10	-	Normal Gland
H11	-	Normal Gland
H12	-	Normal Gland
H13	-	Normal Gland
H14	-	Normal Gland
H15	-	Normal Gland
H16	-	Normal Gland
I1	-	Normal Gland
I2	-	Normal Gland
I3	-	Normal Gland
I4	-	Normal Gland
I5	-	Normal Gland
I6	-	Normal Gland
I7	-	Normal Gland
I8	-	Normal Gland
I9	-	Normal Gland
I10	-	Normal Gland
I11	-	Normal Gland
I12	-	Normal Gland

I13	-	Normal Gland
I14	-	Normal Gland
I15	-	Normal Gland
I16	-	Normal Gland
I17	-	-
J1	-	Normal Gland
J2	-	Normal Gland
J3	-	Normal Gland
J4	-	Normal Gland
J5	-	Normal Gland
J6	-	Normal Gland
J7	-	Normal Gland
J8	-	Normal Gland
J9	-	Normal Gland
J10	-	Normal Gland
J11	-	Normal Gland
J12	-	Normal Gland
J13	-	Normal Gland
J14	-	Normal Gland
J15	-	Suspected Colorectal Ca
J16	-	Suspected Colorectal Ca
J17	-	-
